# Provider Preferences, Perceptions, and Barriers to Colorectal Cancer Screening in Federally Qualified Health Centers: A Cross-Sectional Survey

**DOI:** 10.21203/rs.3.rs-9204324/v1

**Published:** 2026-05-04

**Authors:** Kyle A. Burton, Wyatt Stover, A. Michelle Corbett, Allison Antoine, Carrie Stehman, Evan J. Keiser, Noelle K. LoConte

**Affiliations:** University of Wisconsin–Madison; University of Wisconsin–Madison; Center for Urban Population Health; University of Wisconsin–Madison; Center for Urban Population Health; University of Wisconsin–Madison; University of Wisconsin–Madison

**Keywords:** colorectal cancer screening, federally qualified health centers, stool DNA testing, underserved populations, primary care providers

## Abstract

**Background:**

Colorectal cancer (CRC) screening reduces mortality, yet uptake varies by screening modality, provider type, and clinical setting. Federally Qualified Health Centers (FQHCs) care for underserved populations and face unique challenges, including limited resources, diverse care needs, and competing clinical priorities. Despite the importance of screening, provider preferences for CRC screening modalities are not well understood.

**Methods:**

We conducted a cross-sectional survey of primary care providers (physicians, nurse practitioners, physician assistants) at four FQHCs in Wisconsin. Providers were asked to estimate the percentage of average-risk patients to whom they recommended colonoscopy, stool DNA, and FIT/FOBT (fecal immunochemical test/fecal occult blood test) for two age groups (45–49 and 50–75 years), and to identify factors, barriers, and facilitators influencing their screening decisions. Associations between provider characteristics and recommendations were analyzed using chi-square tests.

**Results:**

33 providers completed the survey (MD/DO = 18, NP = 10, PA = 5). FIT/FOBT recommendations for ages 50–75 differed by provider type. MD/DOs recommended FIT/FOBT to fewer than 25% of patients, while advanced practice providers had a more even distribution across modalities (p = 0.025). When NPs and PAs were analyzed separately, differences remained significant (p = 0.032). Colonoscopy recommendations for ages 45–49 were associated with whether the provider had a family member who completed stool DNA (p = 0.0061). Colonoscopy was the most frequently recommended screening modality, followed by stool DNA and FIT/FOBT. Patient preferences and circumstances were ranked highest among factors influencing screening recommendations. Clinic policy, workflow, and length of patient appointments were the least influential factors. Barriers to prescribing stool DNA included prioritizing other acute health issues, inconvenience, and lack of on-site kit distribution. Provider-ranked patient barriers to completion included complicated instructions, lack of understanding, and fear of results. Top facilitators for increasing stool DNA use included patient reminders, point-of-care kit distribution, and streamlined electronic health record ordering.

**Conclusions:**

In FQHCs, provider type influences CRC screening recommendations, but providers often use multiple modalities suggesting they value tailored recommendations. Targeted efforts to reduce electronic health record workflow challenges, improve kit access and distribution, and enhance patient education may increase screening uptake.

## Background

Colorectal cancer (CRC) remains a leading cause of morbidity and mortality in the United States, ranking as the third most common cancer and the second leading cause of cancer deaths globally [1]. CRC mortality is largely preventable through timely screening which enables detection at earlier and more treatable stages. In the case of colonoscopy, prevention also occurs through removal of precancerous polyps. The 5-year survival rate for early-stage CRC is near 90%, compared to 13.3% for late-stage disease [2]. The US Preventive Services Task Force (USPSTF) recommends CRC screening for all average-risk adults aged 45–75, with selective screening for adults aged 76–85 based on individual health and preferences [3].

Several screening modalities are currently recommended for average risk individuals including colonoscopy, stool DNA testing, and FIT/FOBT (fecal immunochemical test/fecal occult blood test) [4]. Colonoscopy is considered the gold standard and is recommended every 5–10 years, but it requires bowel preparation, sedation, transportation, and time off work [5]. Stool DNA testing detects both blood and tumor DNA fragments and is recommended every 3 years, while FIT is recommended annually. Both can be completed at home which may reduce some of the logistical barriers associated with colonoscopy [3, 6, 7]. If either stool-based test is positive, a follow-up colonoscopy is required. These non-invasive options vary in cost, effectiveness, and patient burden, and screening adherence is likely to improve when recommendations align with patient preferences and circumstances [8, 9].

Despite the proven effectiveness of screening, significant disparities in uptake persist, with CRC screening rates among racial and ethnic minorities remaining disproportionately lower [10]. Federally qualified health centers (FQHCs) provide comprehensive primary and preventive care to underserved populations, including low-income individuals, uninsured patients, and racial and ethnic minority groups [11, 12, 13]. They also serve populations with substantial social risk burden, including income below the federal poverty level, lack of insurance, homelessness, and other indicators of social disadvantage [14]. FQHCs also face unique operational challenges, including higher staff turnover, greater reliance on non-physician providers, and high patient volumes that can impede prioritization of preventive services such as cancer screening [11]. Consequently, CRC screening rates at FQHCs (43%) remain far below the national goal of 80% [15].

Given that provider recommendations are a strong driver of patient adherence to screening, understanding how providers in FQHC settings make screening decisions is critical. However, limited data exist on provider preferences for CRC screening modalities in these settings. We therefore surveyed primary care providers at four FQHCs to assess their preferences, perceptions, and barriers related to CRC screening. This provider survey was conducted alongside a companion focus group study examining stool DNA CRC screening acceptability among racially diverse Black/African-American patients at two FQHCs in Wisconsin [16].

## Methods

We surveyed primary care providers (physicians, nurse practitioners, physician assistants) at four FQHCs in Wisconsin to assess how frequently they recommended various CRC screening modalities including colonoscopy, stool DNA, and FIT/FOBT based on factors influencing screening decisions. Forty-nine providers were invited to complete the survey. Participants were recruited via email invitations and at the completion of the survey received a $25 gift card for their participation. Survey data were collected between July 2023 and October 2023. This project was found to be exempt by the University of Wisconsin–Madison Institutional Review Board.

The survey included questions about providers’ personal experiences with CRC screening, prescription frequency of patients by age group, perceived barriers and facilitators to screening, and overall attitudes toward each screening modality. Although recommendation frequency was assessed across all three guideline-recommended modalities, questions regarding barriers, facilitators, and provider attitudes were focused specifically on stool DNA, consistent with the companion focus group study and its emphasis on expanding acceptability data for this underutilized screening option. These screening recommendations represent the self-reported percentage of patients that the provider would recommend a specific screening test (colonoscopy, stool DNA, or FIT/FOBT). Descriptive statistics were computed for all variables. To test the association between provider characteristics (e.g., provider type, age group, personal experience with screening) and the percentage of patients providers will recommend for screening, we conducted a Pearson’s chi-square test of independence for each of the modalities (e.g., colonoscopy, stool DNA, FIT/FOBT) and each patient age group (45–49 years and 50–75 years). For the analysis, the screening recommendation percentages were categorized into four groups: < 25%, 25–50%, 50–75%, and 75–100%. Additional Pearson’s chi-square testing was done to assess the relationship between provider characteristics and attitudes toward stool DNA screening (e.g., perceived effectiveness, understanding of test mechanism, concerns about DNA collection and specimen storage). For all analysis of provider type, we ran two sets of analyses with provider type grouped (MD/DO, PA/NP) and a second analysis as three distinct categories (MD/DO, PA, NP). Results were analyzed using the statistical software R (Version 4.4.3; R Core Team, 2025).

## Results

A total of 33 providers (18 MD/DO, 10 NP, 5 PA) across four separate FQHCs completed our survey (response rate 67%). Provider specialties included family medicine (24), internal medicine (6), geriatrics (1), and obstetrics/gynecology (2).

### Screening Recommendation Patterns

**Figure 1.**
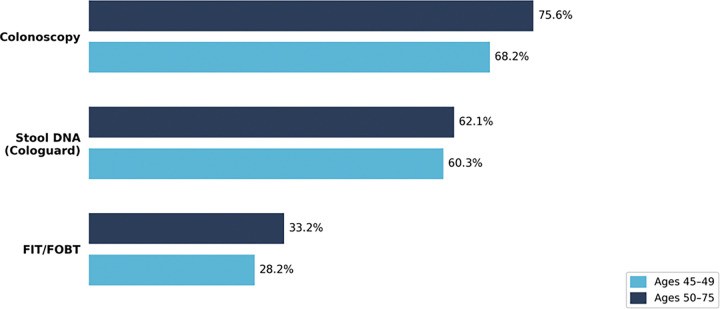
presented by age group and modality Colorectal cancer screening modality recommendations by provider type and patient age group. Bars represent the percentage of providers recommending each modality (colonoscopy, stool DNA/Cologuard, FIT/FOBT) for average-risk patients aged 45–49 and 50–75 years.

Providers were asked to estimate the percentage of their average-risk patients in two age groups (45–49 and 50–75 years) to whom they would recommend each screening modality (colonoscopy, stool DNA, or FIT/FOBT). Colonoscopy was the most frequently recommended screening modality for both age groups, followed by stool DNA, and then FIT/FOBT as shown in Figure 1. Over half of providers reported that they prescribed Cologuard more frequently since the COVID-19 pandemic (51.5%).

### Association Between Provider Type and Screening Recommendations

Provider type was significantly associated with FIT/FOBT recommendation patterns for patients aged 50–75. When grouped as MD/DO versus NP/PA, MD/DOs predominantly recommended FIT/FOBT to fewer than 25% of their patients (14/18, 77.8%), whereas NP/PAs demonstrated a more even distribution across recommendation categories (χ^2^ = 9.267, p = 0.025). This association persisted when providers were analyzed as three distinct categories (MD/DO, NP, PA; χ^2^ = 13.83, p = 0.032). No significant associations between provider type and recommendations were observed for colonoscopy or stool DNA in either age group.

Among patients aged 45–49, colonoscopy recommendation patterns were significantly associated with whether the provider had a first-degree family member who had completed stool DNA screening (χ^2^ = 23.06, p = 0.0061). No other provider characteristics, including personal screening history, age, practice area, attendance at industry-sponsored events, or participation in continuing education, were significantly associated with screening recommendation patterns for either age group.

### Factors Influencing Screening Decisions

**Figure 2.**
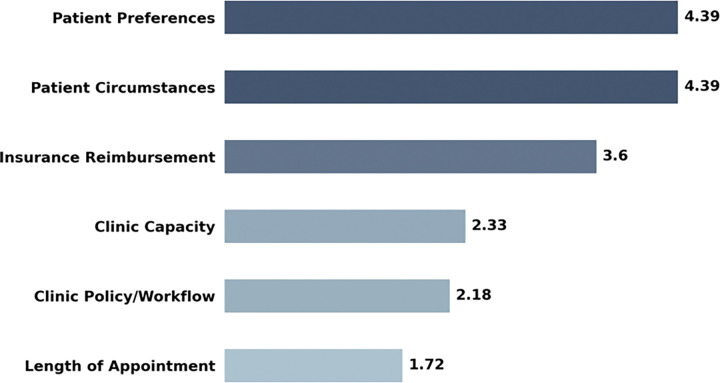
Patient and Clinic Factors Provider-rated importance of patient and clinic factors influencing colorectal cancer screening decisions. Values represent mean importance ratings on a 5-point Likert scale (1 = not at all important, 5 = extremely important), averaged across patient age groups 45–49 and 50–75 years.

Providers ranked patient-centered factors as the most important influences on their screening decisions. Patient preferences (mean 4.42) and patient circumstances (mean 4.36) were rated highest on a 5-point Likert scale for patients aged 50–75, with nearly identical ratings for patients aged 45–49 (4.36 and 4.42, respectively). Insurance reimbursement was rated moderately important across both age groups (3.55 for ages 50–75; 3.64 for ages 45–49). System-level factors were consistently rated as least important, including clinic capacity (2.39; 2.27), clinic policy/workflow (2.12; 2.24), and length of appointment (1.61; 1.82) as shown in figure 2.

### Barriers and Facilitators to Stool DNA Screening

The most frequently reported barrier to prescribing stool DNA was competing clinical priorities, with 60.6% of providers indicating that patients present with other acute or more important health issues that take precedence during appointments. Nearly half of providers (48.5%) reported that patients do not understand how stool DNA testing works. Administrative burden, specifically the cumbersome paperwork and ordering process, was endorsed by 39.4% of providers. Vendor restrictions prohibiting on-site kit storage for point-of-care distribution were reported by 30.3% of providers.

Reminders and support for patients to complete testing (63.6%) and the ability to distribute stool DNA test kits at the point of care (63.6%) were the most reported facilitators for increasing stool DNA prescribing. The ability to prescribe directly from the electronic health record was endorsed by 27.3% of providers, followed by a dedicated clinic workflow for stool DNA (21.2%). These barriers and facilitators are shown in table 1.

## Discussion

This study examined CRC screening preferences, perceptions, and barriers among 33 primary care providers at four FQHCs in Wisconsin. While colonoscopy remained the most frequently recommended modality, providers routinely recommended multiple screening options, and screening decisions were mostly driven by patient-centered factors. These findings have implications for improving CRC screening uptake in FQHCs where rates remain well below national goals [15].

Provider type significantly influenced FIT/FOBT recommendation patterns for patients aged 50–75, with MD/DOs predominantly recommending FIT/FOBT to fewer than 25% of patients while NP/PAs distributed recommendations more broadly. This may reflect differences in training emphasis or comfort with non-invasive screening modalities between provider types and is particularly relevant given the growing role of advanced practice providers in delivering primary care to underserved and socioeconomically disadvantaged populations [17]. The overall preference for colonoscopy, while consistent with national trends [8], is notable given the resource constraints of FQHC settings. Colonoscopy requires extensive bowel preparation, with inadequate preparation occurring in a significant percent of cases and leading to higher complication rates, repeat procedures, and lower patient satisfaction [18, 5]. Stool-based alternatives require less clinic time, no preparation, and can be completed at home [2]. Despite this, FIT/FOBT was recommended to fewer than a third of patients in either age group. This underutilization in a setting where non-invasive options could offer the greatest practical advantage suggests that provider established practice patterns may influence screening decisions.

Patient preferences and circumstances were rated as the most important factors influencing screening decisions, highlighting the importance of informed decision-making when selecting among colorectal cancer screening options [4]. Providers rated system-level factors such as clinic policy and workflow as least important, yet when asked directly about barriers, over 60% cited competing acute health issues, nearly 40% cited administrative burden, and 30% cited inability to distribute kits on-site. This suggests that while providers may not consciously weigh these structural factors when making individual screening recommendations, these barriers still limit stool-based screening uptake in practice. The patient-arm of this study, reported by Keiser et al., found that patient knowledge of CRC and screening options at these same FQHCs was generally low, further underscoring the need for interventions targeting both provider-level and patient-level barriers to screening [16].

The barriers and facilitators identified in our study point toward specific targets for intervention. Patient reminders and point-of-care kit distribution, each reported by nearly two-thirds of providers, represent structural changes that could be implemented without altering individual provider behavior. The finding that nearly half of providers perceived patient comprehension of stool DNA as a barrier highlights a need for improved patient education materials tailored to the diverse literacy levels and language needs of FQHC populations [13]. Streamlining EHR workflows, while reported by a smaller proportion of providers, addresses a modifiable administrative barrier that may contribute to the observed underutilization of stool-based screening. Notably, over half of providers reported prescribing Cologuard more frequently since the COVID-19 pandemic, suggesting that when circumstances favor non-invasive options, providers are willing to shift their practice patterns.

This study does have limitations. The sample of 33 providers across three urban and one rural FQHC limits generalizability, particularly to rural settings where colonoscopy access may be further reduced. Additionally, self-reported survey recommendation percentages may not reflect actual prescribing patterns. Despite these limitations, this study addresses an important gap in understanding how FQHC providers make CRC screening decisions. Future efforts should focus on interventions that reduce administrative burden, improve access to stool-based screening, and enhance patient education. These targeted interventions could help close the gap between current FQHC screening rates and national targets.

## Conclusions

In this cross-sectional survey of providers at four Wisconsin FQHCs, colorectal cancer screening recommendations were shaped by both provider type and patient-centered factors. Although colonoscopy was the most frequently recommended modality, providers commonly used multiple screening approaches, suggesting that individualized recommendations are valued in these settings. Reported barriers to stool-based screening, including competing visit priorities, workflow inefficiencies, limited stool DNA kit access, and gaps in patient understanding, highlight several modifiable targets for intervention. Improving screening delivery in FQHCs may help reduce persistent disparities in colorectal cancer prevention among underserved populations. Efforts focused on streamlining ordering processes, expanding point-of-care kit distribution, and enhancing patient education and support may improve screening uptake and advance equity in colorectal cancer screening.

## Supplementary Material

Supplementary Files

This is a list of supplementary files associated with this preprint. Click to download.

• SupplementaryMaterialProviderSurvery.docx

## Figures and Tables

**Table 1:** Provider Reported Barriers and Facilitators to Stool DNA Screening

Category	Factor	% Providers

**Barriers**		
Clinical Priorities	Patients have other acute/more important health issues	60.6
Patient-Related	Patients don’t understand how the test works	48.5
Administrative	Paperwork/ordering process is cumbersome	39.4
Logistical	Vendor prohibits keeping kits on-site for distribution	30.3

**Facilitators**		
Patient Support	Reminders and support for patients to complete test	63.6
Distribution	Ability to distribute DNA test kit at point of care	63.6
Workflow	Ability to prescribe from electronic health record	27.3
Policy	Dedicated clinic workflow for Cologuard	21.2

Provider-reported barriers and facilitators to stool DNA (Cologuard) screening prescription andpatient completion. Providers could select multiple barriers and facilitators. Percentages represent theproportion of providers endorsing each item.

## Data Availability

The datasets generated and/or analyzed during the current study are available from the corresponding author on reasonable request.

## Abbreviations

CRC: Colorectal cancer
FQHC: Federally Qualified Health Center
FIT: Fecal immunochemical test
FOBT: Fecal occult blood test
FIT/FOBT: Fecal immunochemical test/fecal occult blood test
EHR: Electronic health record
USPSTF: United States Preventive Services Task Force
MD/DO: Doctor of Medicine/Doctor of Osteopathic Medicine
NP: Nurse practitioner
PA: Physician assistant
IRB: Institutional Review Board
COVID-19: Coronavirus disease 2019
